# Polymeric Nanoparticles and Nanogels: How Do They Interact with Proteins?

**DOI:** 10.3390/gels9080632

**Published:** 2023-08-06

**Authors:** Amirhossein Sadeghi, Shadi PourEskandar, Esfandyar Askari, Mohsen Akbari

**Affiliations:** 1 Polymer Laboratory, School of Chemistry, College of Science, University of Tehran, Tehran P.O. Box 141556455, Iran; 2 Department of Chemical Engineering, Razi University, Kermanshah P.O. Box 6718773654, Iran; 3 Biomaterials and Tissue Engineering Research Group, Department of Interdisciplinary Technologies, Breast Cancer Research Center, Motamed Cancer Institute, ACECR, Tehran P.O. Box 1684613114, Iran; 4 Mechanical Engineering Department, University of Victoria, 3800 Finnerty Rd., Victoria, BC V8P 5C2, Canada

**Keywords:** polymeric nanoparticles, nanogels, protein corona, nanoproteomics, disease diagnosis

## Abstract

Polymeric nanomaterials, nanogels, and solid nanoparticles can be fabricated using single or double emulsion methods. These materials hold great promise for various biomedical applications due to their biocompatibility, biodegradability, and their ability to control interactions with body fluids and cells. Despite the increasing use of nanoparticles in biomedicine and the plethora of publications on the topic, the biological behavior and efficacy of polymeric nanoparticles (PNPs) have not been as extensively studied as those of other nanoparticles. The gap between the potential of PNPs and their applications can mainly be attributed to the incomplete understanding of their biological identity. Under physiological conditions, such as specific temperatures and adequate protein concentrations, PNPs become coated with a “protein corona” (PC), rendering them potent tools for proteomics studies. In this review, we initially investigate the synthesis routes and chemical composition of conventional PNPs to better comprehend how they interact with proteins. Subsequently, we comprehensively explore the effects of material and biological parameters on the interactions between nanoparticles and proteins, encompassing reactions such as hydrophobic bonding and electrostatic interactions. Moreover, we delve into recent advances in PNP-based models that can be applied to nanoproteomics, discussing the new opportunities they offer for the clinical translation of nanoparticles and early prediction of diseases. By addressing these essential aspects, we aim to shed light on the potential of polymeric nanoparticles for biomedical applications and foster further research in this critical area.

## 1. Introduction

**Figure 1:**
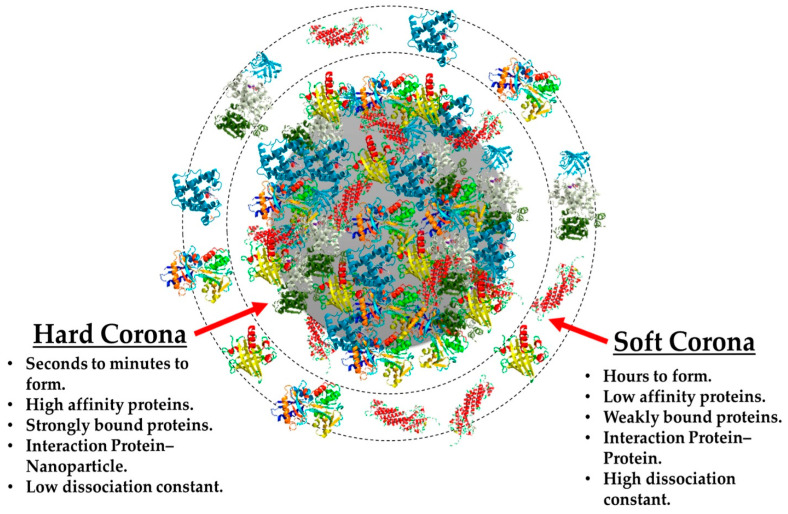
Pattern of a protein corona around a nanoparticle. Reused with permission from MDPI [26].

Fifteen years have elapsed since the term “protein corona” was introduced by Dawson, Linse, and their colleagues [1]. While the adsorption of proteins onto surfaces, such as particles, scaffolds, or implants, has been recognized for quite some time, its true significance in bio-nano research had not been thoroughly examined until the publication of the report. The concept of the protein corona can be likened to the halo of plasma surrounding the sun, where proteins in a solution containing serum form a coating around tiny particles. This analogy draws a parallel between the glowing halo surrounding the sun and the protein coats enveloping particles in serum-containing environments [2]. Recent discoveries have revealed that the formation of a protein corona is driven by thermodynamic reactions in water-based environments, with the goal of minimizing free enthalpy. The process is influenced by underlying physics. Throughout history, substantial efforts have been devoted to understanding the nature and properties of protein coronas, employing a diverse range of models and methods from the fields of chemistry, biology, and material sciences [3]. The exploration of protein coronas holds tremendous potential for diverse biomedical and nanotechnological applications, as it profoundly influences the behavior and interactions of nanoparticles within biological systems. By delving deeper into the complexities of protein corona formation, we can pave the way for more effective and targeted applications in medicine and beyond. In the biomedical domain, nanotechnology plays a crucial role in the development of materials for screening, smart delivery, and medication administration, driving extensive research in this area. When a nanoparticle is introduced into a complex biomedium, it undergoes the Vroman effect, where it initially acquires a temporary or “soft” corona composed of large-sized molecules that are present in high concentrations. Subsequently, the nanoparticle becomes enveloped in a “hard” corona, formed by molecules with a stronger affinity [4]. Figure 1 illustrates this phenomenon conceptually [5]. During protein binding, the enthalpy is lowered, and the nanoparticle’s coat undergoes changes, increasing entropy. These interactions have diverse outcomes, including enhanced bioavailability and dispersion of particles in serum-containing media. Additionally, they can trigger biological processes such as protein misfolding and accumulation, leading to changes in protein structure, and may elicit an immune response in the host to clear the particle. Furthermore, they could conceal the chemical or biological properties of nanoparticles while they circulate in the body [6]. It is essential to address these concerns, whether the nanoparticle serves as a carrier or cargo, as the fate of the nanoparticle is significantly influenced by these biomolecules, irrespective of its intended application. Understanding and controlling these interactions can profoundly impact the effectiveness and safety of nanotechnological interventions. Among nanoparticles, polymeric nanoparticles (PNPs), including nanogels, represent a versatile class of materials with unique properties. Nanogels are a crosslinked form of polymers capable of absorbing and retaining water or water-based fluids [7]. On the other hand, solid polymeric nanoparticles are often fabricated using various techniques such as emulsion polymerization, nanoprecipitation, or solvent evaporation. These solid nanoparticles can exhibit either hydrophilic or hydrophobic characteristics, making them suitable for a wide range of applications in biological environments. Notably, they find use in the smart delivery of therapeutic agents, serve as contrast agents in medical imaging, and act as fillers in tissue engineering applications [8]. Nanogels, characterized by their sub-micron size, can be typically produced using methods like water in oil emulsion or droplet microfluidic technology. Unlike traditional polymeric nanoparticles, nanogels can vary in size due to their swelling and deswelling behavior [9]. One remarkable feature of nanogels is their responsiveness to external stimuli. For instance, temperature- and pH-responsive nanogels can undergo structural changes, alter their surface charge, and modify their affinity in response to changes in temperature or pH [10]. A well-known example is Poly(N-isopropylacrylamide) (PNIPAM), a thermoresponsive hydrogel that experiences changes in its structure and hydrophobicity as the temperature crosses its low critical solution temperature (LCST) [11]. These stimuli-responsive nanogels open up exciting possibilities for controlled drug delivery and targeted therapeutic interventions in biomedical applications.

By harnessing the unique properties and responsiveness of PNPs and nanogels, researchers can develop innovative strategies for more efficient and precisely targeted therapeutic interventions, further advancing the field of nanomedicine. On the other hand, PNPs possess an intrinsic affinity for interacting with solvents, enabling their dispersion or aggregation. The size of a nanoparticle is generally independent of the solution or fluid in which it is dispersed [12,13,14,15]. Both nanogels and PNPs have garnered significant attention as carriers for drug delivery and therapeutic applications. Their ease of fabrication and high throughput, along with their biocompatibility and biodegradability, make them promising candidates for various applications, as extensively covered in numerous reviews [16,17,18]. However, a key challenge associated with the utilization of PNPs and nanogels arises from protein corona formation when they are dispersed in serum-containing media, such as culture media or blood, for applications in therapy or diagnosis [19]. The use of PNPs and nanogels in proteomics has also drawn considerable interest. Extensive literature explores the binding of biomolecules, such as proteins or lipids, with artificial nanoparticles, including PNPs and nanogels [20,21]. In terms of PNPs, important parameters, such as polymer type, and mechanical and chemical characteristics (e.g., surface charge, particle size, and water contact angle), significantly influence the structure of the protein corona [22]. Given the application of PNPs and nanogels in proteomics, a fundamental understanding of protein–nanoparticle interactions, specific adsorption, and protein corona–nanoparticle separation is imperative [23,24,25].

This study aims to collect and examine PNPs and nanogels that have interacted with serum-containing media in various applications to elucidate the physicochemical phenomena occurring in the biological milieu. Initially, we discuss the material type, including the chemical structure and fabrication method, as well as surface properties such as charge and contact angle. Furthermore, we address different surface modifications used to enhance the bio-distribution or targeted adhesion of biomolecules on the surface of nanoparticles, supported by biological factors such as blood rate and temperature. Finally, we explore recent advancements related to protein-corona-isolation methods in the context of proteomics. This comprehensive investigation will contribute to a deeper understanding of the interactions between PNPs and nanogels in biological environments, facilitating their optimal utilization in various biomedical applications.

## 2. Nature and Chemistry of PNPs and Nanogels

Before delving into the materials section, it is imperative to provide an understanding of plasma composition, as it lays the foundation for a more comprehensive analysis of the interactions between PNPs (plasma nanoparticles), nanogels, and proteins. The abundance of proteins in plasma is succinctly summarized in Table 1. Notably, pulmonary-surfactant-associated protein A and serum albumin proteins are particularly prevalent in plasma and demonstrate a strong inclination to interact with nanoparticles. This table illustrates the vast array of proteins in serum, each exhibiting different concentrations and affinities for binding to PNPs and nanogels.

Proteins, which consist of twenty distinct amino acids, encompass a diverse array of properties, ranging from polar and non-polar to hydrophobic and hydrophilic. The primary, secondary, tertiary, and quaternary structures of proteins are constructed through covalent and non-covalent bonding, including electrostatic attraction, hydrogen bonding, and hydrophobic interactions, ultimately resulting in the formation of intricate 2D and 3D protein structures. As chemically active macromolecules, proteins engage in various types of interactions to initiate or catalyze biological processes. When presented with a substrate, proteins readily bind, and likewise, when encountering bare PNPs and nanogels, they readily adhere to their surfaces, employing different bonding techniques for this purpose [27].

The allure of nanoparticles lies in their high surface area and abundant functional groups, rendering them highly attractive candidates for interacting with proteins. Within the field of biomedical engineering, nanoparticles find diverse applications ranging from drug delivery to imaging, thereby necessitating a deep exploration of their interactions with biomolecules and proteins [28]. The inevitability of such interactions stems from the widespread utilization of nanoparticles in this realm.

**Table 1:** Comprehensive list of frequently occurring proteins in Native surfactant (NS) and crude plasma [29]. Reused with permission from ACS.

Native Surfactant.		Crude Plasma	
Proteins	rel. abundance (%)	Proteins	rel. abundance (%)
Pulmonary surfactant-associated protein A	10.19 ± 0.39	Serum albumin	23.15 ± 4.80
Serum albumin	5.77 ± 0.18		
Sodium dependent phosphate transport protein 2B	2.31 ± 0.11	Alpha-2-macroglobulin	11.04 ± 0.98
Tubulin alpha-4A chain	2.27 ± 0.04	Complement C3	8.8 ± 0.39
Fibronectin	2.37 ± 0.07	Lg alpha-1 chain C region	7.7 ± 0.77
Myosin-9	2.09 ± 0.11	Serotransferrin	5.01 ± 0.24
Detected in malignant brain tumor 1 protein	1.99 ± 0.11	Alpha-1-antitrypsin	4.60 ± 0.86
Complement C5	1.85 ± 0.09	Haptoglobin	3.51 ± 0.36
Actin cytoplasmic 1	1.80 ± 0.1	Apolipoprotein A-1	3.26 ± 0.6
Complement C3	1.68 ± 0.19	Lg alpha-1 chain C region	2.42 ± 0.16
Pulmonary surfactant-associated protein B	1.39 ± 0.11	Lg gamma-1 chain C region	2.20 ± 0.25
Lg alpha-1 chain C region	1.28 ± 0.08	Complement C4-A	2.09 ± 0.14
Hemoglobin subunit beta	1.23 ± 0.01	Lg alpha-1 chain C region	2.07 ± 0.31
l-xylulose reductase	1.14 ± 0.06	Lg gamma-1 chain C region	2.05 ± 0.06
Tubulin beta-48 chain	1.02 ± 0.07	Hemopexin	1.79 ± 0.13
Tubulin alpha-1A chain	1.02 ± 0.07	Ceruloplasmin	1.35 ± 0.12
Calcium-activated chloride channel regulator 1	0.96 ± 0.04	Lg lambda-1 chain C regions	0.91 ± 0.04
Polymeric immunoglobin receptor	0.94 ± 0.03	Alpha-1-antichymotrypsin	0.98 ± 0.05
AP-2 complex subunit beta	0.94 ± 0.08	Interalpha-trypsin inhibitor heavy chain-H2	0.88 ± 0.07
Serotransferrin	0.92 ± 0.03	Complement factor H	0.81 ± 0.07
		Ig mu chain C region	0.8 ± 0.08

Among the wide variety of nanoparticles available, including PNPs, metals, metal–organic frameworks, and ceramics, PNPs and nanogels have garnered significant attention in the field of biomedical engineering. This heightened interest can be attributed to their straightforward fabrication methods, biocompatibility, biodegradability, and ease of surface modification, making them particularly promising for various biomedical applications [30].

**Figure 2:**
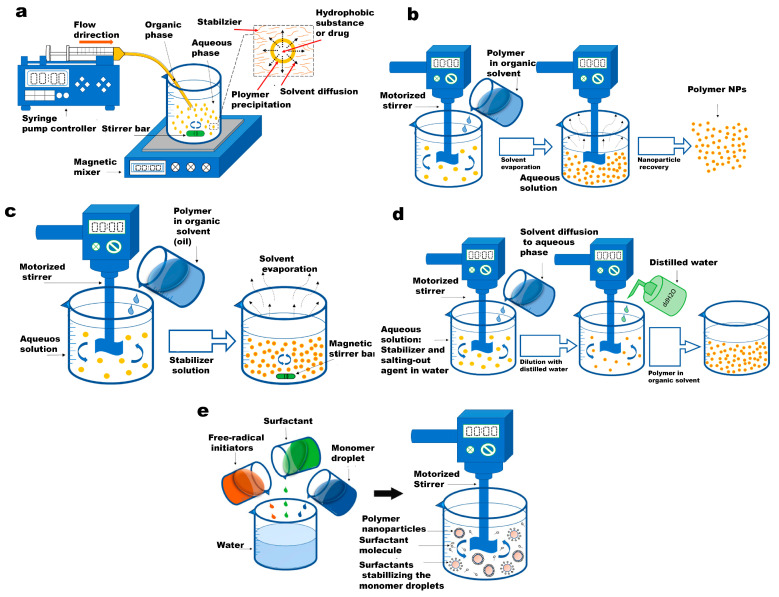
Schematic presentation of conventional polymeric nanoparticle routes. (**a**) The diagram showcases the nanoprecipitation process. The inset, which is an enlarged image, elucidates the formation of nanoparticles (depicted as yellow spheres). This formation is attributed to the disparity in surface tension between the aqueous phase (characterized by high surface tension) and the organic phase (characterized by low surface tension). (**b**) emulsification-solvent evaporation technique. (**c**) emulsification solvent diffusion method. (**d**) salting-out technique. (**e**) emulsion polymerization method. Reused with permission from MDPI [31].

Figure 2 illustrates the conventional fabrication pathways for PNPs and nanogels that are commonly employed in biomedical applications. Additionally, Table 2 provides a comparative analysis of these routes. The synthesis methods can be categorized into two main groups: nanoparticle synthesis from a pre-formed polymer and nanoparticle formation through monomer polymerization. These approaches offer distinct advantages and are selected based on the specific requirements of the intended biomedical application.

In the group of pre-formed polymers, the emulsion preparation phase remains consistent across all methods, while the second phase varies among the techniques. The most commonly used methods for PNP synthesis from a pre-formed polymer solution are nanoprecipitation (NP), emulsification–solvent evaporation (ESE), emulsification–solvent diffusion (ESD), and salting-out (SO) [33]. On the other hand, for monomer polymerization, emulsion polymerization (EP) and mini-emulsion polymerization (MEP) stand out as powerful techniques for nanoparticle synthesis [34]. These approaches enable the fabrication of PNPs and nanogels while allowing control over particle size, polydispersity index, and surface chemistry. This control is achieved through adjustments in prepolymer or monomer concentration, stirring rate, surfactant concentration, and the type of polymerization or crosslinking used. However, it is important to note that batch emulsion-based methods, while suitable for large-scale production, may sacrifice homogeneity in the produced nanoparticles. The fabrication of multilayered nanoparticles using these methods can be challenging due to differences in surface tensions, leading to the aggregation of one material in the core of the nanoparticles. Overcoming these limitations, microfluidic technology offers the potential to generate side-by-side multimaterial nanoparticles, but large-scale production using integrated microfluidic methods requires further advancements in fabrication techniques [35].

**Figure 3:**
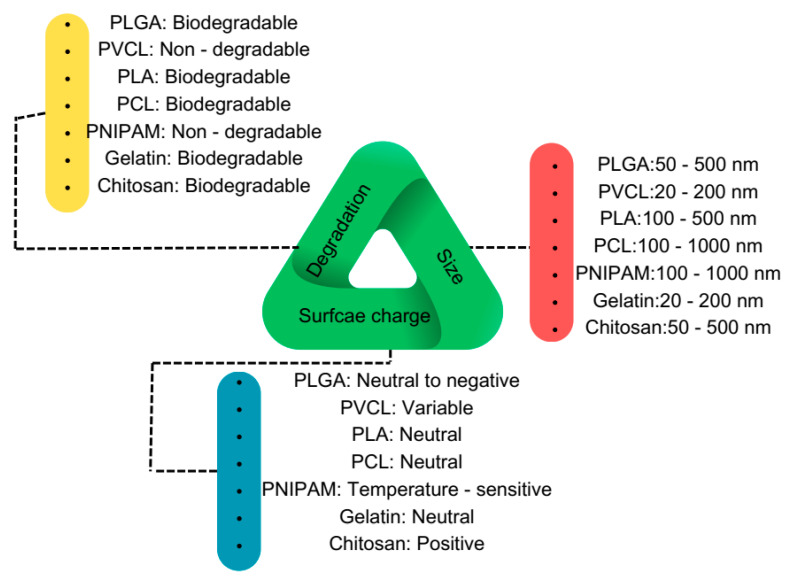
Schematic overview of the most used biocompatible polymers at the nanoscale for biomedical applications.

Regarding material selection, six widely used, biocompatible, and mostly biodegradable synthetic and natural polymers have been chosen: Poly(d,l-lactide-co-glycolide) (PLGA), poly(caprolactone) (PCL), Poly(N-vinyl caprolactam) (PVCL), PNIPAM, Poly(lactic acid) (PLA), gelatin, and chitosan. These polymers serve as source materials for PNP and nanogel fabrication, enabling the study of their chemistry and mechanical properties. Figure 3 presents a triangular schematic, with each line representing one critical feature of PNPs and nanogels. The size of these nanoparticles varies depending on the fabrication method, ranging from 50 nm to 250 nm, which has been extensively utilized for investigating their interaction with proteins.

Regarding surface charge, the focus has been on the bare nanoparticle’s charge, though it is feasible to modify the surface charge by coating the nanoparticles with different materials. Initially, the surface charge is controlled by the charged or functional groups of the polymers and can later be altered to become more negative, positive, or even naturally charged, depending on the study’s objectives. In terms of biodegradability, all polymers except PNIPAM and PVCL are biodegradable and can degrade in physiological fluids and temperatures. In the following sections, we will review the chemical structures and properties of these polymers, as well as the various applications they have been employed for.

### 2.1. Poly(d,l-lactate-co-glycolide)

**Figure 4:**
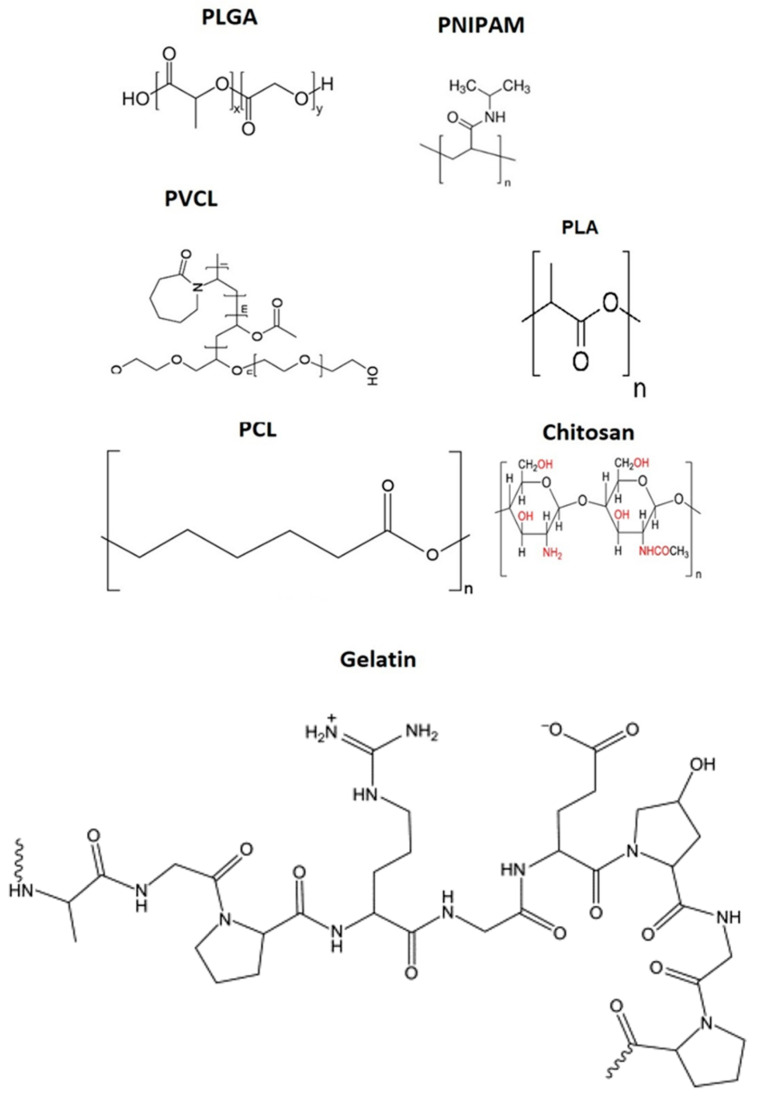
Chemical structure of polymers that are mostly utilized in nanoparticle synthesis for biomedical applications.

The biodegradable system PLGA has proven to be a reliable tool in the development of nanomedicine, as it undergoes degradation in the biological body to form biodegradable metabolites, namely, lactic acid and glycolic acid. Figure 4 presents the chemical structure of PLGA and other polymers commonly used in biomedical applications. Due to the body’s efficient handling of these two monomers, PLGA exhibits extremely low systemic toxicity when used for drug delivery or tissue engineering purposes.

Various techniques, such as emulsification–diffusion, solvent emulsion–evaporation [36], interfacial deposition, and nanoprecipitation [37], have been employed to prepare PLGA nanoparticles. In the emulsification–diffusion technique, PLGA polymers are dispersed in a nonaqueous solvent, isolated in a water-based phase containing an additive, and mixed using a mixer. Subsequently, the obtained polymers are dispersed in a volatile nonaqueous solution, which is then added to a continuously swirling water-based phase containing an emulsifier/stabilizer and subjected to sonication for solvent evaporation. Interfacial deposition techniques have been used to prepare both nanocapsules and nanospheres.

Nanoprecipitation, the process in which tiny particles are formed in an intermediate film of water and water-miscible organic solvent, followed by separation through centrifugation, stands out as the most frequently used method for preparing PLGA nanoparticles. In this method, an acetone-dissolved polymer is added dropwise to a continuously swirling aqueous phase, with or without an emulsifying agent, and the organic solvent is vaporized under low pressure. PLGA nanostructures have found application in the preparation of nanomedicines for chemotherapy, radiotherapy, immunotherapy, and various combinations of these therapies. The effectiveness and release of prepared nanomedicines are significantly influenced by factors such as the decoration of PLGA surfaces, agent-loading techniques, size distributions introduced during formulation, molecular drug weight, and the ratio of lactide to glycoside moieties [36].

Due to their acidic nature, PLGA monomers are not directly suitable for use in medicines or bioactive compounds. To address this challenge, various approaches have been developed, including the use of ingredients like alginate [38], chitosan [39], poly(vinyl alcohol) [40], and others to prepare PLGA nanomedicine formulations. Recognized as a safe material by the US Food and Drug Administration (FDA), PLGA has seen the development and commercialization of numerous medications for a variety of ailments, with several agents effectively loaded into PLGA nanoparticles or bonded to them [41].

Before administering drug-loaded PLGA nanoparticles into the bloodstream, it is crucial to investigate their bio-distribution by studying interactions between PLGA nanoparticles and proteins. When foreign objects are introduced into animal and human serums, the behavior of both changes. Consequently, the introduction of PLGA nanoparticles affects the protein compositions of animal and human serums differently. According to Partikel et al., the protein corona compositions resulting from the incubation of PLGA nanoparticles (214.6 and 221 nm) with animal and human serums are distinct [42]. Sodium dodecyl sulfate–polyacrylamide gel electrophoresis (SDS-PAGE) is one of the powerful methods for studying the nature of the protein corona. Table 3 presents the most abundant proteins exhibiting the strongest tendency for adsorption on the surface of PLGA. Notably, tubulin alpha-4A chain, actin, cytoplasmic 1, and hemoglobin subunit beta are essential proteins showing strong tendencies to interact with PLGA nanoparticles. The composition of proteins on the surface of PLGA nanoparticles mainly depends on the surface chemistry of the nanoparticles [43].

### 2.2. Poly(N-isopropylacrylamide)

In 1986, Pelton and Chibante reported the first surfactant-free emulsion polymerization of PNIPAM at 70 °C, using N,N 0-methylenebisacrylamide (MBA) as a crosslinker and potassium persulfate (KPS) as an initiator. This method involves the formation of nanogel particles through homogeneous nucleation. The breakdown of KPS at elevated temperatures generates sulfate radicals, initiating NIPAM polymerization. As a PNIPAM chain reaches a certain size, it collapses upon itself, leading to the release of precursor particles. This chain collapse occurs because the polymerization temperature is higher than the polymer’s lower critical solution temperature (LCST) [44]. These precursor particles then propagate through various routes, including aggregation with other precursor particles, incorporation into existing particles, collection of increasing radical particles, and further addition of monomers. To produce smaller nanogel particles, it is essential to stabilize the precursor particles early in the process. Since the charge supplied by the initiator fragments is insufficient to stabilize the tiny precursor particles during polymerization, an ionic surfactant can be utilized to provide colloidal stability. Similarly, reducing the surfactant content can lead to the formation of larger particles [45].

Wu et al. were the first to provide transformation vs. time graphs for PNIPAM nanoparticles generated through emulsion polymerization with sodium dodecyl sulfate (SDS) [46]. The polymerization rates were enhanced by temperature and N,N′-methylene-bis-acrylamide (MBA), with MBA being utilized as the crosslinking monomer at a faster rate than NIPAM. Increasing the amount of SDS used in the polymerization resulted in a decrease in particle diameter, suggesting a slight increase in the polymerization ratio with SDS content. The average crosslinking density of the nanogels decreased with transformation, as indicated by the swelling-related findings of these nanogels. This was attributed to the faster metabolism of MBA, leading to the formation of particles with a high crosslinking density [46].

The temperature-sensitive surface of PNIPAM nanoparticles plays a role in modulating the composition of the protein corona. Below the LCST, the solvation of PNIPAM weakens the interactions between proteins and PNIPAM chains, increasing the energy required for the formation of firmly bound proteins (hard corona) surrounding the nanoparticles. At this temperature, the proteins weakly attach to the nanoparticles (soft corona). By contrast, when PNIPAM is in the hydrophobic state (T > LCST), the strength of protein–PNIPAM chain interactions increases, leading to a decrease in the energy required to build a hard corona surrounding the nanoparticles, resulting in increased protein adsorption. To establish the change in the outer-layer properties of PNIPAM leading to the development of the protein corona, the quantities of proteins on magnetic microparticles modified with PNIPAM were evaluated during incubation in human plasma at 25 °C and 37 °C for 2 h. T > LCST induced a somewhat higher density of proteins on the micro-beads (105 µg/mL) than LCST (90 µg/mL). The temperature during the incubation of PNIPAM-based surfaces in protein mixtures may influence the quantity of proteins attached to the surfaces [47].

### 2.3. Poly(N-vinylcaprolactam) (PVCL)

In 1999, Gao et al. reported the pioneering synthesis of poly(N-vinylcaprolactam) (PVCL)-based nanogels at 70 °C and 108 °C through emulsion polymerization of VCL. They utilized MBA as a crosslinker, SDS as a surfactant, and either KPS or tert-butyl hydroperoxide (TBHPO) as an initiator [48]. As the synthesis temperature exceeded the LCST of PVCL chains, particle formation occurred through homogeneous nucleation, similar to PNIPAM-based nanoparticles. Additionally, they observed that PVCL-based nanogels exhibited behavior similar to PNIPAM-based nanogels regarding swelling and de-swelling.

Subsequently, Imaz et al. investigated the kinetics of PVCL-based nanogel production. They conducted emulsion polymerization of VCL using MBA as a crosslinker, SDS as a surfactant, and KPS as an initiator at 70 °C, and compared the resulting product with PNIPAM- and PVCL-based nanogels [49]. When KPS was used without a buffer, the pH of the reaction medium decreased, leading to an increase in the hydrolysis percentage of VCL. These findings suggested a competition between the spread and hydrolysis of VCL. To maintain a constant neutral pH value in the reaction medium and prevent hydrolysis, a buffer was introduced. In the case of PNIPAM-based nanoparticles, the results indicated that MBA interacted more quickly than VCL in terms of partial conversion development. The crosslinker was entirely consumed within 8 min in the buffered reaction, while the full conversion of VCL took 90 min. However, when poly(ethylene glycol) diacrylate (PEGDA) was used as the crosslinker instead of MBA due to differences in the hydrophilicity of PEGDA and MBA, limited VCL conversion and somewhat faster crosslinker consumption were observed [50].

### 2.4. Poly(lactic acid)

**Figure 5:**
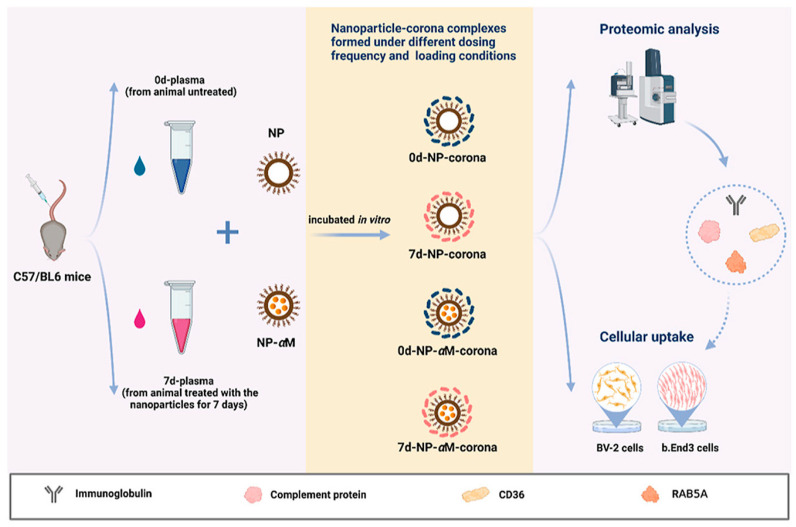
Drug loading on a PEG-PLA nanoparticle and its application in biomedicine [53]. Reused with permission from Elsevier.

A schematic illustration of the PLA-based nanoparticles utilized in nanoproteomics is presented in Figure 5. PLA is a non-toxic and biodegradable material that naturally breaks down into monomeric lactic acid units as part of carbohydrate metabolism in vivo. Simple emulsification–solvent evaporation (ESE) has been employed to prepare PLA nanoparticles. Additionally, the salting-out (SO) process has been used, which involves separating a water-miscible solvent from an aqueous solution using a salting-out agent like magnesium chloride or calcium chloride [51]. The significant advantage of the SO technique lies in its ability to reduce protein encapsulant stress.

However, a major limitation arises when PLA nanoparticles are administered into the bloodstream, as they tend to develop a protein corona on their outer layer. To address this issue, researchers such as Macedo da Luz have investigated the biocompatibility and internalization efficiency of PLA nanoparticles of different sizes (diameters of 63 and 66 nm) in human lung epithelial A549 cells. They observed that the diameter of the nanoparticles increased when introduced into the culture medium, providing evidence of protein corona formation [52].

To regulate the composition of the protein corona on the surface of PLA nanoparticles, PEGylation, a process involving the linking of poly(ethylene glycol) on the nanoparticle surface as a coating, has been utilized [53,54,55].

### 2.5. Polycaprolactone

PCL has garnered considerable interest in drug administration due to its degradation through the hydrolysis of ester bonds in biological media. Its properties are particularly intriguing for the advancement of implanted devices because PCL degrades at a slower rate than polylactide [56]. To prepare PCL nanoparticles, the SD and SE techniques have been utilized [57]. Similar to other materials, the physicochemical properties of PCL nanoparticles play a crucial role in protein adsorption on their surfaces. In a comparative study, PCL nanoparticles exhibited a stronger tendency to bind to human serum albumin than PLGA nanoparticles [58]. To improve the long-term distribution of PCL nanoparticles and minimize protein binding, surface modifications have been performed. Huang et al. reported that collagenase IV and clusterin-modified PCL-PEG nanoparticles resulted in weak protein corona formation on the outer surfaces of the nanoparticles [59].

### 2.6. Chitosan

Chitosan is a modified natural carbohydrate polymer obtained by partially N-deacetylating the biopolymer chitin, produced by crustaceans. Four approaches are employed to form chitosan nanoparticles: ionotropic gelation, micro-emulsion, emulsification–solvent diffusion, and polyelectrolyte complex formation [60]. Ionotropic gelation involves electrostatic interactions between the amine group of chitosan and the negatively charged groups of polyanions, such as tripolyphosphate. Chitosan is dispersed in acetic acid with or without a stabilizing agent. The addition of the polyanion and mechanical churning leads to the spontaneous formation of nanoparticles. In the micro-emulsion process, a surfactant is dispersed in a nonaqueous solution like n-hexane. Chitosan in an acidic solution and the crosslinker are then added to the surfactant/hexane combination under continuous mixing [60].

Due to their positive surface charge and other physicochemical properties, chitosan nanoparticles are widely used as nanoparticles or as a coating layer for other PNPs and nanogels [61]. However, according to Almalik et al., bare chitosan nanoparticles exhibit high immunogenicity. To address this, they investigated the effect of coating chitosan nanoparticles with hyaluronic acid (HA) and alginate on the protein corona formed on their surfaces. The HA-based surface coating significantly reduced protein adsorption and resulted in the lowest immunogenicity compared to the alginate-coated and bare chitosan nanoparticles. Interestingly, selective protein adsorption was observed on the HA-coated chitosan nanoparticles, including two unique anti-inflammatory proteins (ITIH4 and AGP), which were not detected in the alginate-coated and bare chitosan nanoparticle samples. On the other hand, pro-inflammatory protein (clusterin) was selectively adsorbed on the surface of the alginate-coated and bare chitosan nanoparticles [62].

### 2.7. Gelatin

Gelatin finds common usage in food, pharmaceutical, and regenerative medicine applications due to its cost-effectiveness, safety, and easy biodegradability [63]. As a polyampholyte, gelatin contains charged and hydrophilic groups. The mechanical characteristics, expansion behavior, and thermal properties of gelatin are known to be influenced by crosslinking levels [64]. Gelatin nanoparticles can be prepared using desolvation/coacervation [65] or emulsion methods. In the coacervation process, positively and negatively charged macromolecules aggregate in the dense concentrated polymer phase at the bottom and the clear solution phase above, respectively. Alcohol and, in some cases, natural salts can be used as tools for particle size quality control in coacervation and turbidity/crosslinking.

Meghani et al. [66] reported that when positively charged gelatin–oleic nanoparticles were incubated with human serum albumin under shear stress, the growth of protein corona on the outer layer of the nanoparticles increased their size from 300 nm to 341 nm and changed their zeta potential to negative. Furthermore, the inhibitory concentration of drug-loaded gelatin nanoparticles increased to a higher level after incubation with human serum albumin. Nguyen et al. studied gelatin–oleic nanoparticles to assess the effect of the BSA-formed protein corona on the biological efficacy of the nanoparticles; they found that BSA had a similar effect on the size, surface charge, biodistribution, and cellular uptake of gelatin–oleic nanoparticles [67].

## 3. Circulation Time and Clearance of Nanoparticles

Nanoparticles play a crucial role in efficiently transporting medicines to affected tissues, but they need to evade clearance through the reticuloendothelial system (RES) and avoid being trapped in the lung, liver, or spleen [68]. The size of nanoparticles significantly affects their opsonin binding and, consequently, their susceptibility to phagocytosis. Macrophages find it easier to take up larger nanoparticles compared to smaller ones due to the challenging geometric arrangement required for successful complement activation on the more strongly curved surfaces of smaller particles. Small nanoparticles (10–20 nm) can bypass tight endothelial connections in various organs and are swiftly expelled through the kidney’s glomeruli. Additionally, they have the ability to re-enter the circulatory system, reducing the amount of chemotherapy drug remaining in tumor tissue [69]. On the other hand, large particles (>1 µm) also have a high clearance rate. In physiological conditions, large particles tend to agglomerate and are mechanically trapped by capillaries. From a hydrodynamic perspective, as particle size increases, momentum forces become dominant, increasing the likelihood of wall collisions. This results in rapid absorption by microphysiological systems (MPS) and substantial accumulation in the liver, spleen, and, to a lesser extent, the bone marrow. When particle sizes fall between these two extremes (20 nm and 1 µm), the effects of all these clearance processes diminish, leading to a considerable increase in circulation duration [70].

For nanoparticles to be effectively delivered systemically, their diameters should ideally range from 20 to 100 nm. Nanoparticles with diameters exceeding 20 nm can prevent filtration by the kidney [71], but they should not exceed 100 nm to avoid specific sequestration by sinusoids in the spleen and liver fenestra, which have diameters of 150–200 nm [72]. Additionally, the size of fenestrations in tumor vasculature limits the accumulation of nanoparticles in tumors under the enhanced permeability and retention effect. The size of these fenestrations varies depending on various factors such as the type of cancer, stage of illness, location in the body, and host species [73]. These findings underscore the importance of particle size in determining their efficacy in drug delivery. Protein corona formation can increase the hydrodynamic size of nanoparticles, making them more susceptible to elimination by the immune or other clearance systems.

## 4. Effect of Nanoparticle Size and Biological Conditions on Their Interaction with Proteins

Previous investigations have predominantly utilized nanoparticles that are considerably larger in size than serum proteins, leading to the characterization of nanoparticles as the “carriers” and the proteins as the “cargo” [74]. It has been postulated that this understanding of protein corona design and biological effects could be extended to ultra-small nanoparticles with diameters smaller than 10 nm [75]. Before delving into the interaction of proteins with ultra-small nanoparticles, we will briefly review the concept of single-chain PNPs and the existing methods for synthesizing such nanoparticles.

Depending on the targeted particle size and desired applications, several synthetic techniques have been developed. The top-down technique involves mini- and micro-emulsion systems to produce crosslinked nanoparticles through nanoemulsified solvent methods, resulting in nanoparticles with diameters smaller than 50 nm. Conversely, a novel bottom-up strategy based on the crosslinking of self-assembled block copolymer micelles has yielded a broad spectrum of particles. However, the synthesis of extremely small particles requires more intricate methods, such as dendrimers, which range in size from 1 to 10 nm [76].

The intra-molecular chain collapse method facilitates the production of higher quantities of nanoparticles in the 3–15 nm size range. The number of functionalities is regulated by the structure of the linear precursor, resulting in the extremely accurate fabrication of PNPs within a particularly restricted size range. Although the synthesis is carried out in an extremely diluted setting (0.5 µg L^−1^) at the outset, the use of highly effective crosslinking processes and permanent addition systems allows for the fabrication to be carried out at higher concentrations [77]. Stimuli-sensitive nanoparticles have been reported to be produced using this technique, involving supra-molecular reactions that facilitate swelling of the structures in response to UV irradiation and pH changes [78,79,80].

**Figure 6:**
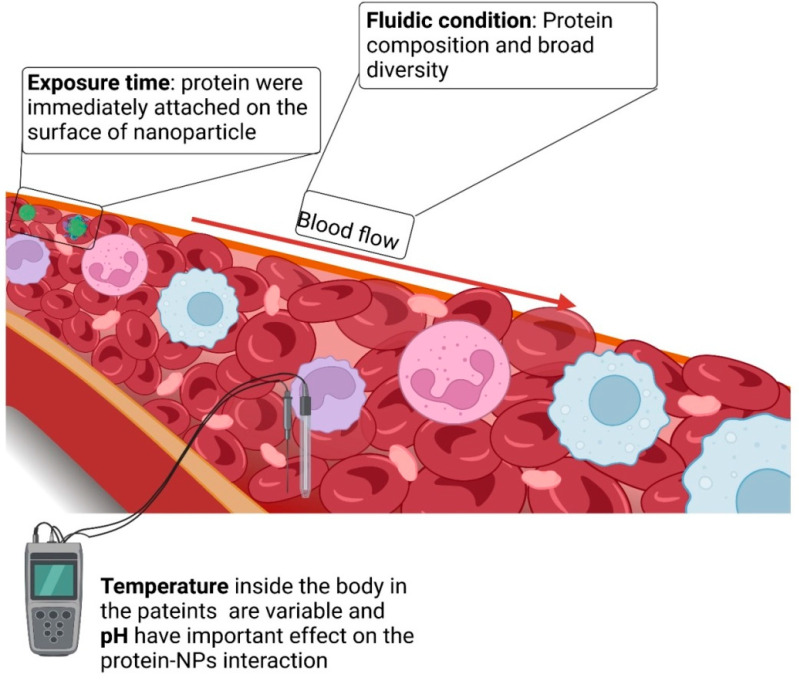
Biological parameters influence nanoparticle–biomolecule interactions.

As an important parameter in protein corona assembly, PNP or nanogel size affects the nature and composition of the protein corona. Moreover, the biological milieu around the nanosystem also influences the composition of the protein corona (Figure 6). Consequently, the composition of the protein corona may differ depending on individual genotypes and environmental factors, such as geographic location and food intake. Furthermore, even within the healthy temperature range (35–40 °C), temperature fluctuations might change the composition of the protein corona. These results imply that the potential impact of temperature in the specific body location should be considered at all times [81].

Blood flow is another important physical element that must be considered. Blood moves at a constant rate, ranging from 0.085 cm.s^−1^ in capillaries to 10 cm.s^−1^ in arteries, and a maximum of 60 cm.s^−1^ inside the aorta. Protein binding generally strengthens with increasing blood flow. However, the exact underlying cause is unknown, and it is speculated that the enhanced protein–blood interactions result from increased interactions between biological molecules and the nanostructures. Additionally, rapid flow rates induce shear stress on the outer layer of nanostructures, preventing the easy deposition of proteins and making it easier for them to connect with others that possess stronger affinity. For example, this might lead to the enrichment of proteins on the nanoparticles, such as plasminogen and other proteins. Similarly, blood flow could alter the conformational shape of unbound proteins, such as plasminogen or the von Willebrand factor (a protein that alleviates the dormant factor VIII blood-clotting protein).

Diverse conformations can lead to variable interactions with nanosystems, and consequently, different protein corona compositions [82]. Furthermore, illnesses can alter the protein corona of a nanosystem. Researchers have investigated the effect of protein shortages on the remodeling of the protein corona on PS nanoparticles due to common illnesses and medical situations [83]. They concluded that the protein corona differed significantly across disorders, emphasizing the relevance and complexity of recreating the optimal biological environment.

## 5. Effect of Nanoparticle Surface on Their Interaction with Proteins

Proteins constitute the majority of bonded biomolecules on the outer layer of nanoparticles in a serum-containing medium, but recently, traces of lipids have also been discovered [84]. Both protein–nanoparticle adsorption affinities and protein–protein interactions influence the binding of proteins on the outer layer of nanoparticles. Proteins with a hard corona are composed of firmly attached proteins that are difficult to detach, whereas proteins with a soft corona are composed of lightly adsorbed proteins. Moreover, soft and hard coronas can be distinguished based on their interchange times. Hard coronas exhibit substantially longer exchange durations, ranging from several hours to many days [26]. According to one theory, proteins in the hard corona completely connect with the surface of the nanomaterial, whereas protein–protein interactions occur at the interface between the soft and hard coronas. Even at the minimum concentration of biomolecules, the corona layer provides total surface coverage. By contrast, the adsorbed corona does not entirely obscure the outer layer of nanoparticles [82].

The thickness of the protein corona can be influenced by several factors, including protein content, particle size, and particle surface characteristics. Proteins in plasma have hydrodynamic sizes ranging from 2 to 20 nm, and the formed protein layer is extremely thick, comprising multiple layers of linked proteins rather than just one. Simberg et al. introduced the concept of the protein corona [85]. According to this concept, the protein corona contains “primary binders” that directly recognize the outer layer of nanoparticles and “secondary binders” that interact with the primary binders through protein–protein interactions. The secondary binders may modify the activity of the primary binders or “cover” them, thus inhibiting their contact, which is crucial for the physiological response [86].

**Figure 7:**
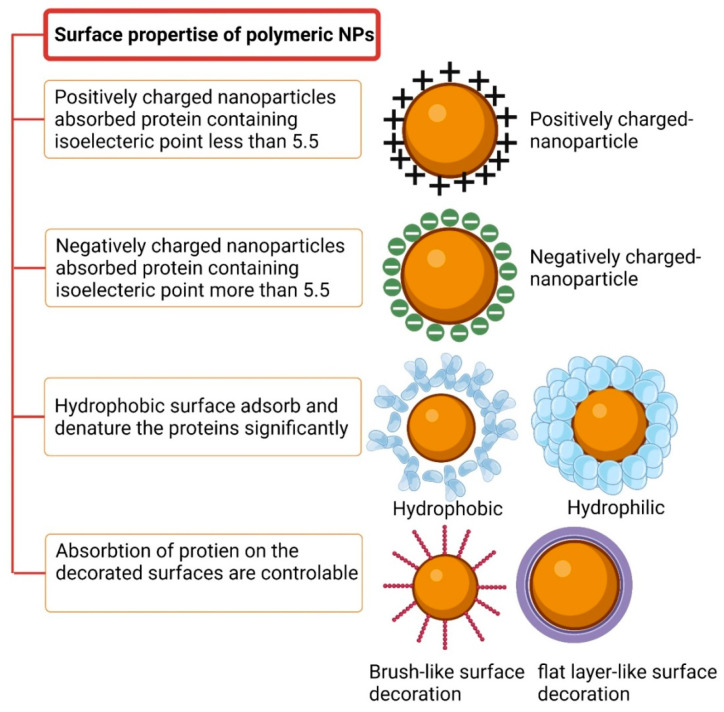
The surface of a polymeric nanoparticle manages the protein–NP interaction.

Walkey and Chan reviewed a group of plasma proteins called adsorbomes that are commonly found in the protein coronas of nanomaterials [87]. This list is expected to grow in the future with increasing research on the topic. Based on nearly 20 years of research, a “normal” plasma protein corona consists of approximately 2–6 proteins that are bound abundantly, along with numerous additional proteins that are bound with lower abundances. Most nanomaterials are connected to only a small fraction of plasma adsorbomes, and only a small fraction of the adsorbomes is related to abundant nanomaterials. The “Vroman effect” defines the competition of proteins on the confined surface of nanoparticles, taking into account the effects of the incubation period, protein concentration, and protein-to-nanoparticle surface adsorption affinity [88]. Figure 7 illustrates the effects of surface properties on the protein corona. Another crucial factor related to the associated proteins is the positive or negative charge, which is typically analyzed in terms of zeta potential. Protein adsorption is enhanced when nanoparticles are efficiently charged (either more positively or more negatively). The isoelectric point (IEP) corresponds to the pH value at which a particle has no charge. Positively charged nanoparticles strongly bind to biomolecules with IEP values lower than 5.5, such as BSA, whereas nanoparticles with negative surface charge tend to bind to proteins with IEP values higher than 5.5, such as IgG [89]. Serum protein adsorption changes as the surface charge density of PNPs increases. Gessner et al. observed this effect using negatively charged PNPs [90]. Additionally, Bradley et al. discovered C1q binding to anionic liposomes and observed plasma protein adsorption on cationic lipid-based vesicles. These phenomena may occur due to electrostatic interactions between positively charged serum biomolecules and lipids. The adsorbed proteins can potentially be denatured by the surface charge [91].

The quantity of adsorbed protein and the nature of the protein-adsorbed layer are influenced by hydrophobicity [92]. Proteins are more readily adsorbed on hydrophobic surfaces than on hydrophilic surfaces, leading to an increased opsonization rate of hydrophobic nanoparticles. Hydrophobic or charged surfaces absorb and denature larger amounts of protein compared to neutral and hydrophilic surfaces. For instance, hydrophobic polystyrene nanoparticles with a negative charge density absorb more protein from serum than polystyrene nanoparticles composed of hydrophilic polymers [93]. Even though the affinity of proteins for both types of nanoparticles is approximately the same, hydrophobic nanoparticles absorb more albumin molecules than their hydrophilic counterparts [94]. This suggests that hydrophobic copolymer nanoparticles have a greater number of protein-binding sites, possibly because the hydrophobic polymer chains cluster together, forming separate “islands” that act as protein-binding sites.

An important discovery reported by Moghimi and Patel was that cholesterol-rich liposomes adsorb smaller amounts of biomolecules than cholesterol-free liposomes [95]. The binding of blood proteins to liposomes composed of neutral saturated lipids with carbon chains above C16 has been found to be more effective than liposomes composed of neutral saturated lipids with carbon chains below C14. Plasma proteins, especially IgG and albumin, exhibit a stronger affinity for hydrophobic domains. Consequently, as charge density and hydrophobicity increase, the affinity of proteins for nanomaterials with a homogeneous surface chemistry also increases.

**Figure 8:**
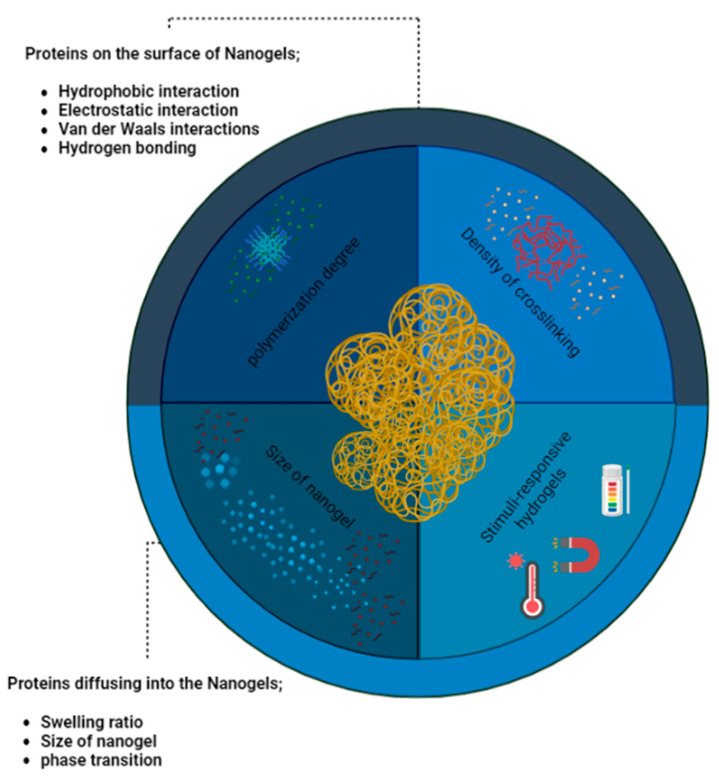
Nanogels encounter a diverse array of proteins and biomolecules, their interplay being heavily influenced by the surface and bulk characteristics of these nanoscale gel-like structures. The intricate interplay between nanogels and the surrounding media is dictated by their finely tuned surface and bulk properties, imparting a crucial role in the regulation of their interactions.

As can be shown in the Figure 8, he significance of surface chemistry, encompassing charge, hydrophilicity, and interactions with biomolecules, cannot be overstated in hydrogels and nanogels concerning the uptake of proteins and biomolecules [96]. The degree of crosslinking in hydrogels provides various means to regulate interactions with biomolecules. Notably, the water uptake capacity of hydrogels enables interactions with proteins and biomolecules through water diffusion channels. The size of these channels or a lower degree of crosslinking can facilitate protein diffusion, potentially influencing bulk properties like mechanical characteristics and degradation rate through specific protein binding. For instance, in a study by Khoury et al., the authors found that the swelling of a pH-responsive hydrogel led to an increase in protein diffusion within the hydrogel [97].

The diffusion of proteins in hydrogels is primarily governed by hydrophobic and van der Waals interactions, while hydrogen bonding has a lesser impact on protein dispersion in water and protein diffusion within the hydrogel. Additionally, the degree of crosslinking and polymerization can affect the interaction between nanogels, polymeric nanoparticles, and biomolecules. Uncrosslinked monomers, positioned in the hydrophobic region of the polymer, may exhibit reduced tendencies to dissociate in the final hydrogel, thereby influencing electrostatically driven biomolecule interactions. Consequently, the manipulation of hydrogel and nanogel properties, encompassing surface chemistry and crosslinking, offers promising avenues for controlling biomolecule uptake and interactions [98].

## 6. Surface Decoration

The protein corona of a nanosystem may be influenced by surface modifications on nanoparticles, such as antibodies, proteins, and peptides [99,100,101,102]. Surface modifications, including ligand features (nature, size, conformation, etc.), can alter the initial protein corona structure [103,104]. For example, Zhang et al. investigated the effects of three distinct ligands on the development of the protein corona (two different 1-kDa peptides and transferrin; MW: 77 kDa). In vivo, the ligands particularly affected hemoglobin, Apo E, Apo A-IV, clusterin, and hemoglobin subunit 2. Furthermore, protein adsorption caused a loss of targeting capacity in several of these cases [100]. However, the targeting abilities of other systems remained unaffected by their protein coronas. In the case of nanosystems coated with fluorescent dyes, a change in the protein corona was observed. Surface modification of PS nanoparticles with rhodamine B caused a threefold decrease in fibrinogen adsorption and a twofold increase in IgG adsorption. Consequently, the absorption of this nanosystem on human HL60 cells significantly decreased [105].

In the following section, the popular surface-modification systems relevant to nanoparticles are discussed from the viewpoint of reducing or engineering protein adsorption.

### 6.1. Poly(ethylene glycol) (PEG)-Based Decoration

The grafting of PEG moieties onto nanomaterials is a widely employed technique for increasing circulation time and reducing phagocyte clearance [106]. PEG chains create a strongly hydrophilic environment that prevents particles from interacting with even the most hydrophobic compounds. Consequently, the nanoparticle surface acts as a steric barrier, impeding the approach of molecules. The PEG stealth effect is determined, in part, by the number of PEG chains per unit surface area of the nanosystem (PEG density) and the PEG chain length (PEG MW). When the PEG concentration is low, the most stable shape is the mushroom configuration, which allows for stronger interactions with proteins. Conversely, at higher PEG concentrations (>7–20 chains/cm^2^), PEG forms a brush-like structure, making its interaction with proteins more challenging [107].

PEG molecules with molecular weights (MW) higher than 5 kDa exhibit similar stealth capabilities. At lower MW values (5 kDa), the stealth effect intensifies as the PEG chain length increases, as longer chains create a stronger barrier between the nanoparticle surface and unbound proteins. The stealth effect is observed at an MW of 2 kDa. These effects have been demonstrated for several types of nanosystems, including PGMA, PLA, PLGA, and PCL nanoparticles. However, it is essential to note that PEGylation does not entirely eliminate protein adsorption. On PEGylated surfaces, certain proteins, especially dysopsonins, can still be identified. In other cases, such as with PS nanoparticles, these proteins may play a more critical role in minimizing cell absorption than PEGylation itself [107].

Despite these advantages, PEGylation has certain drawbacks. For example, it may hinder the recognition of ligand-functionalized nanosystems by their target receptors. To reduce the occurrence of this phenomenon, various strategies have been investigated. One approach involves introducing targeting ligands to the final part of the PEG chain to impart flexibility to conjugated molecules and reduce protein adsorption, thereby simplifying targeting. Another method involves the regulated in vivo breakdown of the PEG-NP linker and the subsequent separation of PEG molecules, facilitating the exposure of targeted ligands. Enzymatic or pH-dependent cleavage of PEG linkers can reveal hidden ligands. PEGylation selectively enriches specific proteins in the protein corona of nanoparticles, including clusterin and apolipoprotein A-I [108].

### 6.2. Polyphosphoester-Based Decoration

In the realm of nucleic and teichoic acids, polyphosphoesters (PPEs) possess distinct chemistry that enables control over their hydrophilicity and degradation. PPEs with pronounced hydrophilicity exert an effect similar to that of PEG. The stealth properties of PPE and PEG were assessed on PS nanoparticles with amine-functionalized surface groups. When compared to PEGylation (around +10 mV), PPEylation caused a remarkable reversal and substantial shift in the outer-layer zeta potential of the particles (from +46 to approximately 10 mV). Notably, the PPEylated corona lacked a significant amount of Apo A-I, unlike the PEGylated nanoparticles [109].

### 6.3. Polysaccharide-Based Decoration

Nanoparticles were coated with polysialic acid (PSA), a hydrophilic and negatively charged polysaccharide commonly found on mammalian cell surface proteins, which effectively repels negative proteins [110]. PSA is a naturally occurring substance with semi-biocompatibility and limited toxicity. Currently, phase II clinical trials are underway for a PSA derivative in lung cancer vaccines and hemophilia A therapy, showing promising potential [111]. Notably, in the case of hemophilia A therapy, conjugating recombinant factor VIII to PSA resulted in a circulation time similar to that of the already-commercialized PEGylated factor VIII. Additionally, in HeLa cells, PSA-coated exosomes were observed to be recognized and absorbed by sialic-acid-binding immunoglobulin-like lectin receptors during protein interactions [112].

PLGA nanoparticles coated with sialic acid have shown a reduction in the production of pro-inflammatory cytokines [113]. The use of hyaluronic acid (HA) has received approval for various applications, including in the eyes, nose, lungs, and parenteral nutrition. Studies have demonstrated that HA coating prevents CS nanoparticles from aggregating upon interaction with serum and also reduces pro-inflammatory protein adsorption. Nevertheless, the shielding effect of HA has been a subject of debate, as some researchers argue that its interaction with proteins and the subsequent immunogenicity may be influenced by its molecular weight (MW) [62].

### 6.4. Artificial Protein Corona Decoration

Naked nanoparticles possess a high affinity for biomolecules, as they can easily bind to the nanoparticle surface through various bonding methods. However, pre-coating nanoparticles with biomolecules and proteins reduces the rate of interaction between the nanoparticles and proteins, as compared to bare nanoparticles. When nanoparticles are pre-suspended with specific proteins, a distinct protein corona is formed in vivo, rich in those particular proteins, which subsequently influences processes such as cellular absorption by specific cells. For instance, pretreated tiny PS particles modified with dysopsonins Apo A-IV and Apo C-III exhibit reduced cellular uptake, whereas coating the same particles with 2-glycoprotein 1 yields the opposite outcome. These findings suggest that Apo A-IV and Apo C-III may be beneficial for enhancing specific uptake, while clusterin coating effectively prevents nanoparticles from adhering to non-specific targets [114].

In the context of PLGA particles, researchers have reported that small PLGA particles adhere more strongly to endothelial cells in the presence of IgA- and IgM-depleted serum, which does not occur in the presence of these specific proteins [115]. However, a drawback is observed, as biomolecules may form novel interactions with additional plasma proteins, resulting in the broadening of the initial protein corona. For example, Mirshafiee et al. coated nanoparticles with globulins to increase macrophage absorption [116]. However, the adsorption of extra proteins hindered these globulins from interacting with macrophage Fc receptors. Therefore, any in vitro evaluation of pre-coated nanoparticles should be preceded by their incubation in plasma.

## 7. Protein–Corona Separation Routes

In this section, we delve into the clinical applications of the protein corona and its implications for diagnoses. In the preceding sections, we discussed the chemistry and fabrication methods of PNPs. While these steps have been extensively employed to enhance the bio-distribution of nanoparticles, they also hold significant potential for disease diagnosis and prediction. To advance the concept of a personalized protein corona, researchers must effectively separate adsorbed proteins from nanoparticles. The analysis of the protein corona involves several key steps: fabrication and decoration of particles, gathering the physiological medium, incubating particles with the medium under time- and temperature-dependent conditions, separating incubated particles, isolating unbound proteins or biomolecules, and utilizing proteomics approaches to identify protein composition. Common methods for isolating nanoparticles coated with a protein corona include centrifugation, size-exclusion chromatography, and magnetic isolation. Among these, centrifugation stands out as the most widely used technique for collecting corona-coated nanoparticles.

### 7.1. Centrifugation-Based Approach

In the centrifugation-based approach, separation is achieved by using varying concentrations of nanoparticles and biomolecules in solutions. Centrifugation-based techniques have been employed to explore particle–protein interactions. However, the main disadvantage of these methods is the potential for false-positive results. For instance, biomolecules, such as proteins or protein complexes, that are adsorbed by nanoparticles might settle together with the nanoparticles and their corona during centrifugation. Conversely, false negatives can occur when the centrifugation pressure causes the proteins to separate from the nanoparticle–corona combination.

To address this issue, it is crucial to determine the optimal number of washing stages and the appropriate duration of centrifugation required to effectively separate a specific particle–biomolecule combination from a biomolecule-concentrated medium. However, it appears that this improvement is seldom implemented or, at least, not adequately documented in relevant research papers [54].

### 7.2. Magnetism-Based Approach

The utilization of magnetic force represents a second method for isolating nanoparticles from their corona. Magnetic techniques offer a simpler and faster strategy for isolating compatible nanoparticles [117]. Generally, iron oxide is employed to impart magnetic characteristics to nanoparticles. Iron oxide nanoparticles hold significant promise as tools for tumor detection and can be further modified with polymers or ceramics to create nanocomposites that blend the capabilities of both phases [118]. Nevertheless, in certain cases involving iron oxide nanoparticles, centrifugation techniques are still utilized, occasionally even for comparison. For example, Bonvin and colleagues simultaneously employed both magnetic and centrifugation isolation methods for the same nanoparticles [119]. Various experimental configurations are known, such as the one published by Luborsky and Drummond, which incorporates magnetic columns and magnetic gradients and has been adapted for nanotechnology-based investigations [120].

It is believed that the structure of the nanoparticle–protein corona complex is less influenced by magnetic forces compared to centrifugation. However, agglomeration is more likely to occur as particle size increases. Therefore, magnetic separation is not recommended for nanoparticles with diameters larger than 10 nm. In such cases, increasing the centrifugation speed during multistep purification may be more suitable for larger particles. One significant advantage of magnetic separation lies in its ability to reduce false-positive proteins arising from aggregation under centrifugal pressures. Additionally, it minimizes protein loss after multiple washing steps. While post-magnetic separation washing steps are necessary, the amount of particles lost in each step may decrease [54]. However, it should be noted that magnetic particles may interfere with other analytical procedures, and the range of nanoparticles and chemical compounds that can be effectively separated using this method is limited.

### 7.3. Chromatography-Based Approach

Chromatographic procedures are less commonly employed than the methods mentioned above due to their time-consuming and costly nature, as well as their limitations in analyzing a smaller number of samples. Additionally, these methods may be incompatible with a wide range of nanoparticle sizes, polydispersity, and nanoparticles that adhere to the column material. However, they do offer the advantage of investigating individual protein–nanoparticle association/dissociation rates and attraction, as well as collecting diverse portions of an experiment with minimal disruptive effects on particle–protein complexes [121].

**Figure 9:**
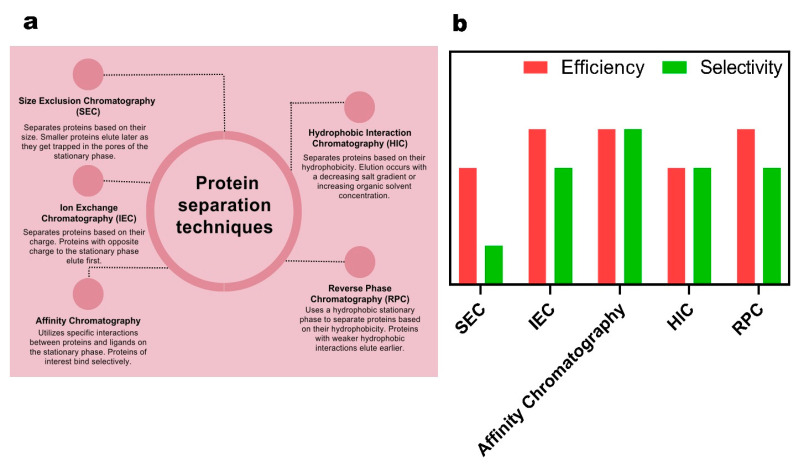
(**a**) Schematic presentation of the most common methods in chromatography-based protein analysis. (**b**) Quantitative bar chart demonstrating the efficacy and selectivity of chromatography-based protein analysis methods.

In Figure 9, the definitions and functioning of chromatography-based methods for protein analysis are illustrated, and their efficiency and selectivity are presented using bar chart plots. More than five methods have been discovered that rely on the surface charges, size, and hydrophobicity of proteins, and their contributions to proteomics science are vital. Size exclusion chromatography (SEC), a commonly used method in proteomics, separates proteins based on their size and molecular weight. It operates by allowing smaller proteins to penetrate and interact with the porous stationary phase, while larger proteins elute faster as they bypass the pores, offering medium efficiency and low selectivity [122]. Ion exchange chromatography (IEC) in proteomics leverages the differences in charge between proteins. It separates proteins based on their affinity for oppositely charged resin beads, enabling the purification and separation of proteins according to their net charge. IEC exhibits high efficiency and medium selectivity [123]. Affinity chromatography in proteomics exploits the specific interactions between a target protein and a ligand immobilized on a chromatography matrix. This allows for the selective purification and isolation of the target protein from a complex mixture of proteins. The target protein binds to the ligand with high affinity, while non-specific proteins are washed away, resulting in the purification and enrichment of the desired protein of interest. This method offers high efficiency and high selectivity [124]. Hydrophobic interaction chromatography (HIC) in proteomics capitalizes on the varying hydrophobicity of proteins to separate them based on their interactions with a hydrophobic stationary phase. This allows for the purification and analysis of complex protein mixtures. Proteins with higher hydrophobicity tend to bind more strongly to the stationary phase, while less hydrophobic proteins elute earlier, resulting in separation based on their hydrophobic properties. HIC provides medium selectivity and efficiency [125]. Reversed-phase chromatography (RPC) in proteomics employs a nonpolar stationary phase and a polar mobile phase to separate proteins based on their hydrophobicity. Proteins with higher hydrophobicity interact more strongly with the stationary phase, resulting in delayed elution time compared to proteins with lower hydrophobicity. RPC offers high efficiency and medium selectivity for protein analysis [126].

## 8. Nanoproteomics

Despite the groundbreaking discoveries made by the Human Genome Project (HGP), which unveiled thousands of protein-encoding genes within the human body, only a third of these genes have been verified at the protein level [127]. The study of the proteome in the biological milieu commonly employs 2D gel electrophoresis and mass spectrometry. However, two major challenges in effective proteome investigation persist: the separation of proteins of interest from complex mixtures and low protein concentrations [128].

**Figure 10:**
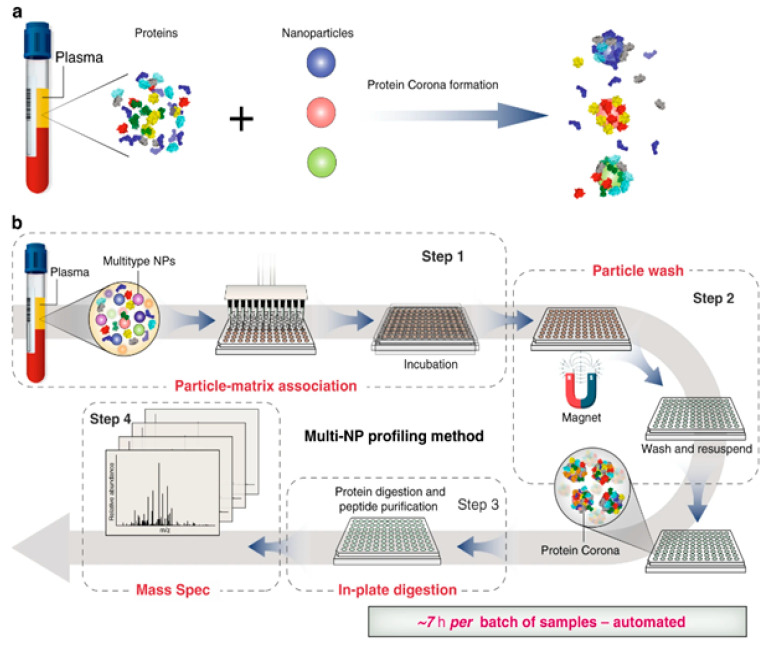
Schematic illustration of automated nanoproteomics [83]. Reused with permission from Nature. (**a**) Incubation of nanoparticles with plasma and protein corona formation. (**b**) automated multi-NP profiling method and Nanoproteomics.

To address these challenges, the use of nanoparticles in proteomics has given rise to a rapidly developing exploration domain known as nanoproteomics, providing a viable solution [129]. Figure 10 depicts a conceptual illustration of nanoproteomics. This novel approach offers advantages such as low sample and material consumption and real-time integrated assessment within a rapid timeframe [130]. Nanoproteomics is emerging as an essential tool for identifying and discovering novel molecular-level objects, thanks to the distinctive properties of nanoparticles, including customizable exterior characteristics and ease of removal from solutions. For instance, polystyrene nanoparticles have been employed in proteomics and diagnosis. When mixed with sera from patients with hypercholesterolemia, diabetes, or rheumatism, polystyrene nanoparticles measuring 100 nm in diameter exhibited significant size changes from 18 nm to 44 nm [131].

Utilizing magnetic particles individually or in composite forms with polymers shows promise in nanoproteomics due to minimal protein loss during magnetization. Blume et al. introduced a fast, detailed, and accurate nanoproteomics protocol for profiling plasma proteomes using multi-protein corona nanoparticles, which is expected to pave the way for innovative studies [83]. From a chemical standpoint, magnetic nanoparticles can enhance the efficiency of the protein corona isolation process. Intriguingly, magnetic-nanoparticle-based nanocomposites, particularly PNPs, have been extensively employed in isolating circulating cancer cells.

Seyfoori et al. made significant strides in the field of nanoproteomics by developing a thermo-responsive nanogel comprising PNIPAM and magnetic nanoparticles to isolate cancer cells from the blood [132]. The initial tier of nanoproteomics involves engineered nanoparticles designed to adsorb specific proteins and biological compounds. Notably, magnetic nanoparticles have demonstrated the magneto-thermal effect in some cases, where a change in the magnetic field occurs under heat. Zhang et al.’s findings revealed that inducing local heat treatment using a magnetic field and magnetic nanoparticles allows for control over the composition of the protein corona. This intriguing feature, with further optimization, holds promise for better control in nanoproteomics research [133].

Magnetothermal regulation of protein corona composition presents another captivating area of research with the potential to yield specific biomarker collections [134]. Magnetic nanoparticles can convert an external alternating magnetic field into heat through hysteresis losses, forming the cornerstone for various applications in stimuli-responsive drug deliveries and imaging [135]. In the context of the protein corona, two key parameters affect the outcome: particle concentration and time of exposure. Particle concentration influences the rate and degree of locally produced heat, directly impacting nanoparticle–protein interactions. The critical factor controlling the heat produced during the experiment is the time of exposure. Locally induced heat plays a crucial role in maintaining specific proteins and detaching linked proteins.

For instance, Zhang et al. utilized iron oxide nanoparticles to investigate how magnetothermal regulation influences the types of proteins on the nanoparticle surface. They demonstrated that localized thermal treatment can function as an annealing treatment, serving as a tool to selectively interact with specific types of proteins and form the protein corona layer [133].

## 9. Conclusions and Future Challenges

This review delved into the intricate interactions between proteins and nanogels and polymeric nanoparticles in protein-contacting environments like blood, serum, or cell cultures. To begin with, the conventional concepts of hard and soft coronas formed on nanoparticle surfaces were introduced. This was followed by a comprehensive discussion of fabrication methods of nanogels and PNPs, serving as drug carriers or nanoparticles to interact with serum. Critical factors such as size, surface charge, and hydrophobicity were thoroughly explored, as they significantly influence the composition of the protein corona. Surface modification techniques like PEGylation, enhanced hydrophilicity, and artificial protein corona addition were investigated for targeted protein corona formation. Protein separation and nanoproteomics emerged as intriguing applications of the protein corona, with promising potential for disease diagnosis and treatment. The review covered centrifugation, magnetism-, and chromatography-based techniques for isolating protein corona nanoparticles. While this review aimed to address the primary needs in the field of protein separation and nanoproteomics, several challenges still require consideration.

In the synthesis and fabrication realm, the use of stimuli-responsive PNPs and nanogels offers the potential for specific protein adsorption and desorption. The regulation of surface hydrophilicity in thermo-responsive materials through temperature holds promise for protein separation applications. Additionally, the synthesis of ultra-small nanoparticles (sub-10 nm) through single-chain polymer reactions presents attractive properties due to their high surface area, but their effects on the protein corona formation mechanism remain an unknown aspect. Scaling up the production of ultra-small nanoparticles poses a challenge, necessitating innovative fixes or regulations of existing methods.

Evaluating the performance of nanoparticles in whole blood or human serum is crucial for identifying their strengths and weaknesses. Sustained microfluidic separation methods may prove useful in this regard. Size-based or spiral geometries for trapping protein coronas offer promising approaches, fostering collaboration among chemists, material scientists, and biological researchers.

The incorporation of magnetic nanoparticles in PNPs and nanogels holds potential for their efficient use in solution without protein loss and non-specific separation. The integration of new genomics, proteomics, high-throughput methods, and nanotechnologies with clinical correlations may be the key to effective personalized therapy. The convergence of nanoproteomics and personalized medicine represents the fusion of protein and nanoparticle technology, promising groundbreaking advancements in the field.

## Figures and Tables

**Table 2:** Most commonly used technologies for producing polymeric nanoparticles [32]. Reused with permission from Elsevier.

Preparation Method		Unique Features	Polymeric Carrier	Cargos Packaged
Batch-mode preparation	Nanoprecipitation Emulsion/Evaporation Dialysis	Does not require special apparatus or devices. Poor micro mixing, leading to relatively low drug loading efficiency and capacity. Low uniformity in size and shape High batch to batch variability Challenging to scale up. Particles of well-defined size and shape (sphere, rods and cubs), high loading efficiency, time consuming process.	Block copolymers, polyelectrolytes, polymer-drug conjugates	Small molecules drugs, nucleic acids, proteins, antibodies, contrast agents
	Layer-by-layer assembly		Polyelectrolytes, proteins, hydrogen-bond forming, polymers, template needed	Small molecules drugs, nucleic acids, proteins, Antibodies, contrast agent
Flow-based preparation	Flash Nanoprecipitation	Well-defined micromixing environment High drug loading efficiency High yield of formulations with uniformity in size Excellent control over particle size Continuous, low-cost, and scalable process	Block copolymer, homopolymer, polymer sugar conjugates	Small molecule drugs. Contrast agents. Semiconducting polymers. Gold NPs.
	Microfluidic devices	Enable high-throughput screening of NPs High reproducibility Challenging to scale up	Block copolymer, Polyelectrolytes	Small molecule drugs, nucleic acids, proteins, antibodies, contrast agents
Lithography-based preparation	PRINT	Particles of well-defined size and shape (sphere, rods and cubs), Can be scaled up. Limited choice of cargoes for loading	Polymers, proteins	Small molecule drugs. SiRNA Contrast agents

**Table 3:** The most abundant proteins interacting with the PLGA NPs [29]. Reused with permission from ACS.

Protein	[%]	STD
Tubulin alpha-4A chain	9.29	±0.56
Actin, cytoplasmic 1	8.04	±0.29
Hemoglobin subunit beta	6.03	±0.28
I-xylulose reductase	5.28	±0.31
Tubulin beta-4B chain	3.68	±0.3
Tubulin alpha-1A chain	3.59	±0.19
Detected in malignant brain tumor 1 protein	3.25	±0.34
Tubulin beta chain	3.23	±0.21
Pulmonary surfactant-associated protein A	2.79	±0.55
Mysoin-9	2.28	±0.11
BPI fold-containing family B member	2.27	±0.14
Fibronectin	2.24	±0.23
Serum albumin	2.03	0.12
Glyceraldehyde-3-phosphate dehydrogenase	1.96	±0.14
Elongation factor 1-lpha-1	1.73	±0.08
ADP-ribosylation factor 1	0.98	±0.06
Tubulin beta-2B chain	0.83	±0.08
Retained dehydrogenase 1	0.83	±0.08
Complement C5	0.83	±0.02
Calcium-activated chloride channel regulator	0.79	±0.06
EH domain-containing protein 2	0.76	±0.04
Fatty acid synthase	0.69	±0.03
Protein-glutamine gamma-glutamyltransferase 2	0.65	±0.03
Complement C3	0.64	±0.1
Pynuvate kinase PKM	0.63	±0.04

## Data Availability

The raw/processed data required to reproduce these findings can be shared upon request.
